# Chromosome-Borne CTX-M-65 Extended-Spectrum β-Lactamase–Producing *Salmonella enterica* Serovar Infantis, Taiwan

**DOI:** 10.3201/eid2908.230472

**Published:** 2023-08

**Authors:** Ying-Shu Liao, Hsiao-Lun Wei, Hung-Chih Kuo, Bo-Han Chen, You-Wun Wang, Ru-Hsiou Teng, Yu-Ping Hong, Jui-Hsien Chang, Shiu-Yun Liang, Chi-Sen Tsao, Chien-Shun Chiou

**Affiliations:** Centers for Disease Control, Taichung, Taiwan (Y.-S. Liao, H.-L. Wei, B.-H. Chen, Y.-W. Wang, R.-H. Teng, Y.-P. Hong, J.-H. Chang, S.-Y. Liang, C.-S. Tsao, C.-S. Chiou);; National Chiayi University, Chiayi, Taiwan (H.-C. Kuo)

**Keywords:** *Salmonella enterica*, bacteria, serovar Infantis, chromosome-borne, CTX-M-65, extended-spectrum β-lactamase, multidrug resistance, chickens, plasmid, chromosome, DNA transposable elements, Taiwan

## Abstract

A CTX-M-65‒producing *Salmonella enterica* serovar Infantis clone, probably originating in Latin America and initially reported in the United States, has emerged in Taiwan. Chicken meat is the most likely primary carrier. Four of the 9 drug resistance genes have integrated into the chromosome: *bla*_CTX-M-65_, *tet(A)*, *sul1*, and *aadA1*.

*Salmonella enterica* serovar Infantis is one of the most common *Salmonella* serotypes (*1*); it is frequently isolated from humans and animals, particularly from poultry (*2*). An increasing incidence of *Salmonella* Infantis infections has been reported in the United States (*3*), accompanied by emergence and spread of an extended-spectrum β-lactamase CTX-M-65‒producing *Salmonella* Infantis clone in humans, food animals, and retail chicken (*4**,**5*). The clone probably originated in South America because it was initially discovered in persons who had traveled back from Peru, Bolivia, Ecuador, and Chile since 2012 (*5*). Domestically acquired infections were not identified in the United States until 2014 (*5*).

This clone is characterized by having a D87Y mutation in the *gyrA* gene and carrying multiple resistance genes, including *aph(**4**)-Ia*, *aac(**3**)-IVa*, *aph(3′)-Ic*, *bla*_CTX-M-65_, *fosA3*, *floR*, *dfrA14*, *sul1*, *tet(A)*, and *aadA1*, located in 2 distinct regions of a pESI-like megaplasmid (*4*). The CTX-M-65‒producing clone has been reported mostly in South America, North America, and some countries in Europe (*4**–**12**)*.

In Taiwan, *Salmonella* Infantis is not a common cause of human salmonellosis, accounting for only 0.61% (246/40,599) of all *Salmonella* isolates collected during 2004‒2022. *Salmonella* Infantis isolates collected during 2004‒2019 showed a low level of antimicrobial drug resistance (Appendix Table 1). However, in 2021, we identified that 7 of 14 *Salmonella* Infantis isolates from patients who had salmonellosis were multidrug-resistant (MDR), and in 2022, MDR strains accounted for 55% (21/38) of the *Salmonella* Infantis isolates recovered that year.

The 28 patients who contracted MDR *Salmonella* Infantis were from diverse age groups and geographic locations, and none of them had a history of international travel. During 2021 and 2022, the COVID-19 pandemic restricted travel abroad. We report a CTX-M-65‒producing *Salmonella* Infantis clone in Taiwan.

## The Study

We performed clustering analysis on pulsed-field gel electrophoresis (PFGE) patterns of *Salmonella* Infantis isolates, which showed that the MDR isolates recovered in 2021 and 2022 clustered closely together in a distinct group (Appendix Figure). Antimicrobial drug susceptibility testing showed that the MDR isolates had resistance to ampicillin, cefotaxime, ceftazidime, nalidixic acid, ciprofloxacin (intermediate susceptibility), gentamicin, chloramphenicol, sulfamethoxazole, trimethoprim, and tetracycline (Appendix Figure). The resistance profile closely resembled that of the widespread CTX-M-65‒producing *Salmonella* Infantis clone (*5*).

We isolated *Salmonella* bacteria from retail raw chicken meat sold in 12 supermarket stores in Taichung City in 2022 to investigate the source of MDR *Salmonella* Infantis. All chicken meat samples were sourced from domestic farms. *Salmonella* bacteria were isolated from 191 (65.6%) of 291 chicken meat samples. A total of 379 *Salmonella* isolates were recovered from the 191 samples (1‒2 isolates from each *Salmonella*-positive sample).

Of the 379 isolates, 68.1% (258) were identified to be *Salmonella* Infantis, followed by *Salmonella* Kentucky (17.2%), *Salmonella* Brancaster (2.6%), *Salmonella* Goldcoast (2.6%), *Salmonella* Agona (2.4%), *Salmonella* Enteritidis (2.1%), and 6 other serovars (5.0%). Of the 191 samples, 11% were found to be contaminated with a mixture of *Salmonella* serovars. The 258 *Salmonella* Infantis isolates had 28 PFGE patterns, among which the 6 most common patterns were also observed in the MDR isolates from humans (Appendix Table 2). We performed a clustering analysis of PFGE profiles, which showed that the 258 *Salmonella* Infantis isolates from chicken meat, 28 MDR isolates from humans, and 1 isolate from a diseased pig recovered in 2022, were grouped in a common cluster (data not shown).

We conducted whole-genome sequencing of 51 *Salmonella* Infantis isolates from humans, chickens, and a pig by using the Illumina sequencing platform (https://www.illumina.com) to investigate drug resistance genetic determinants, plasmid incompatibility types, and their genetic relationships. Our analysis showed that all 51 *Salmonella* Infantis isolates belonged to sequence type 32, and 18 MDR *Salmonella* Infantis isolates recovered from humans, chickens, and a pig in 2021 and 2022 had a D87Y mutation in *gyrA*, along with an IncFIB plasmid and 4 common resistance genes: *aadA1*, *bla*_CTX-M-65_, *sul1*, and *tet(A)* (Appendix Table 3). In addition, 15 of the 18 *bla*_CTX-M-65_–carrying isolates had 5 other drug resistance genes: *aac(**3**)-IVa*, *aph(3′)-Ia*, *aph(**4**)-Ia*, *dfrA14*, and *floR*. Two of the isolates had 4 of the 5 drug resistance genes, and 1 did not have any of the 5 genes.

**Figure 1:**
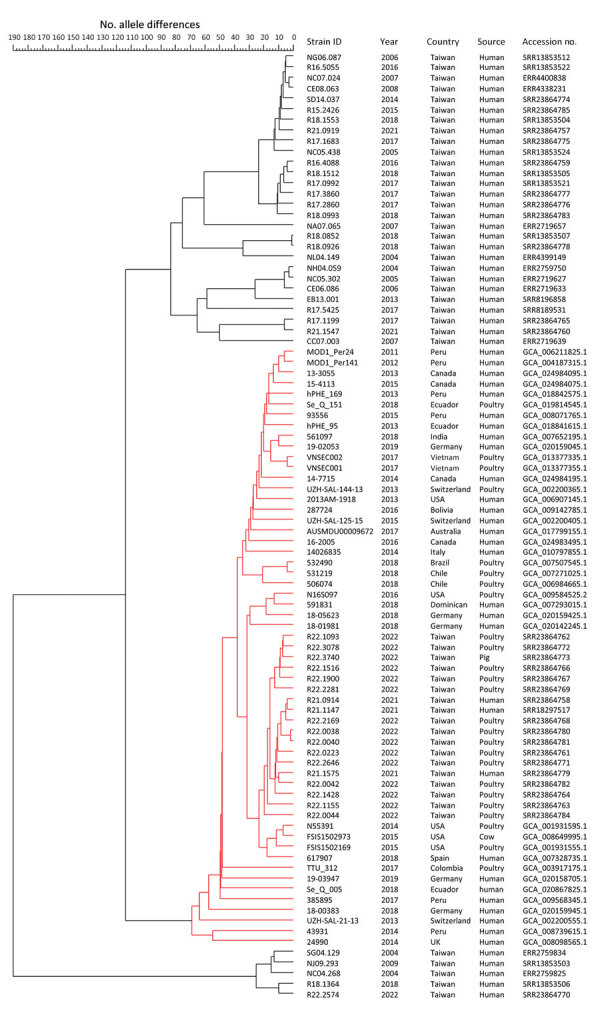
Core genome multilocus sequence typing tree and relevant information for investigation of chromosome-borne CTX-M-65 extended-spectrum β-lactamase–producing *Salmonella enterica* serovar Infantis, Taiwan. The cluster highlighted in red consists of *bla*_CTX-M-65_–carrying strains. GenBank accession numbers are shown. ID, identification.

We conducted clustering analysis of core genome multilocus sequence typing profiles, which showed that the *bla*_CTX-M-65_–carrying isolates from Taiwan, when compared with non‒*bla*_CTX-M-65_–carrying strains, showed a closer genetic relationship with *bla*_CTX-M-65_–carrying strains reported in North and South America, Europe, Australia, India, and Vietnam (Figure 1).

To investigate the location of drug resistance genes, we performed additional sequencing of 6 *bla*_CTX-M-65_–carrying isolates and 1 pan-susceptible isolate by using the Oxford nanopore sequencing platform (https://nanoporetech.com). This approach provided long sequence reads, enabling us to assemble complete genome sequences. Our analysis showed that all 6 *bla*_CTX-M-65_–carrying isolates from humans, chickens, and a pig had 5 drug resistance genes, *aac(**3**)-IVa*, *aph(3′)-Ia*, *aph(**4**)-Ia*, *dfrA14*, and *floR*, within an ≈195-kb IncFIB plasmid. In contrast, *aadA1*, *bla*_CTX-M-65_, *sul1*, and *tet(A)* were found in an ≈126-kb DNA segment inserted within an ABC-F family ATPase gene in the chromosomes (Appendix Tables 4, 5).

Our investigation suggested that the 195-kb IncFIB plasmids and the 126-kb genomic islands found in the chromosome probably originated from a plasmid similar to pN16S097. This megaplasmid, which has a length of 318,524 bp, was initially detected in a *Salmonella* Infantis strain and has 9 of the mentioned drug resistance genes in 2 distinct regions (*8*).

**Figure 2:**
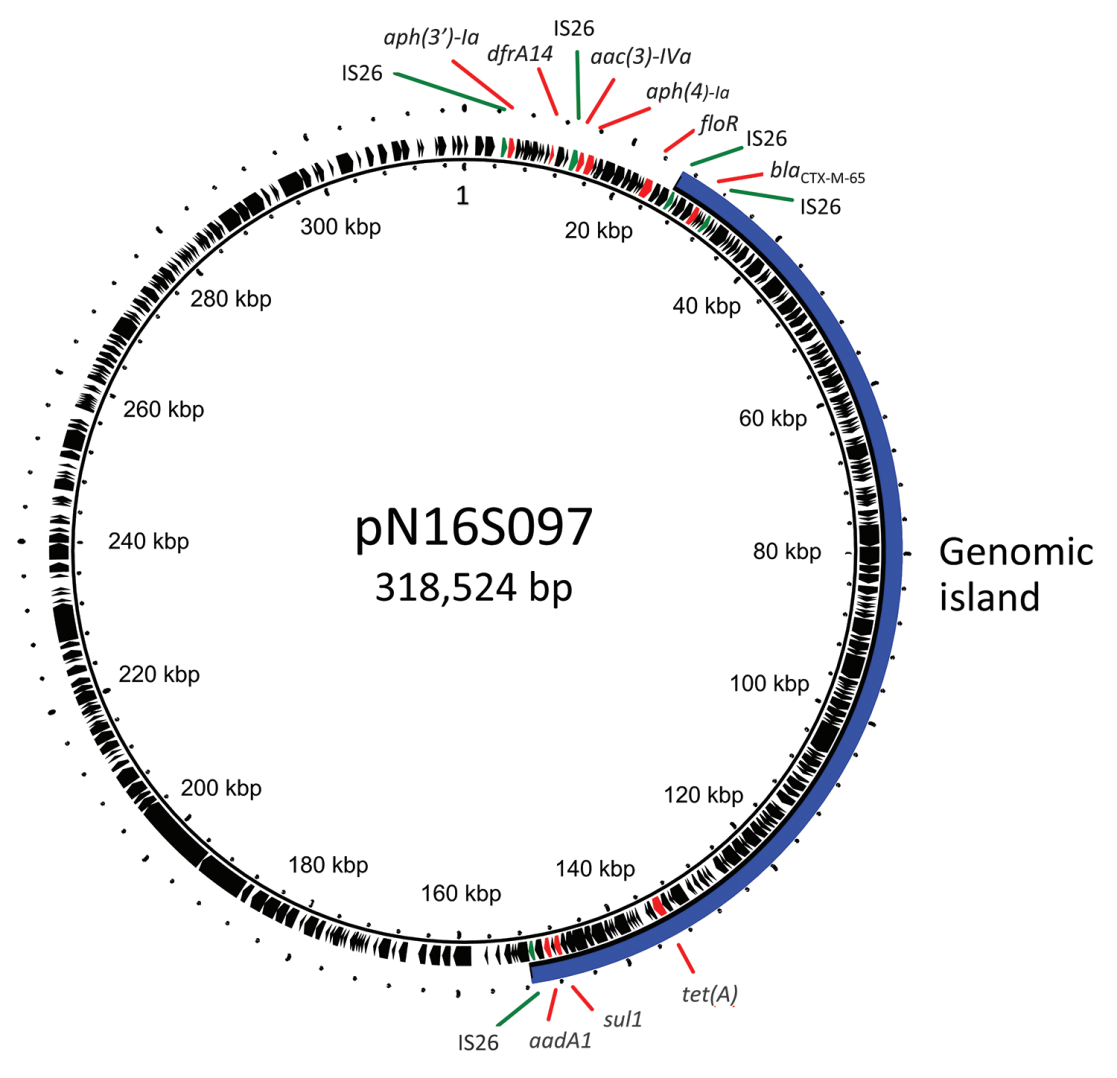
Genetic map of plasmid pN16S097 from investigation of chromosome-borne CTX-M-65 extended-spectrum β-lactamase–producing *Salmonella enterica* serovar Infantis, Taiwan. The locations of antimicrobial drug resistance genes and insertion sequence IS26 are indicated. A 125-kb segment, depicted by a blue solid arc, is translocated into the chromosomes of *bla*_CTX-M-65_–carrying *Salmonella* Infantis strains emerging in Taiwan.

We hypothesize that the 126-kb segment carrying *aadA1*, *bla*_CTX-M-65_, *sul1*, and *tet(A)* might have translocated from a pN16S097-like plasmid into a chromosome through IS26-mediated transposition, resulting in formation of an 8-bp (CCGGAAAG) tandem repeat at the insertion site. This process led to the loss of the megaplasmid, leaving a plasmid of ≈195 kb (Figure 2). Upon analyzing 5,253 genomes of *bla*_CTX-M-65_–carrying *Salmonella* Infantis strains available in GenBank, we did not observe a large DNA segment or a *bla*_CTX-M-65_–carrying segment inserted within an ABC-F family ATPase gene in the chromosomes.

## Conclusions

The *bla*_CTX-M-65_–carrying *Salmonella* Infantis clone, previously identified in South and North America and some countries in Europe, has been detected in Taiwan. Chickens are suspected to be the primary source of *bla*_CTX-M-65_–carrying strains. Many PFGE genotypes have been found among the isolates from retail chicken meat, indicating that the *bla*_CTX-M-65_–carrying *Salmonella* Infantis strains have probably evolved and proliferated on chicken farms, rather than being contaminants from chicken processing plants. Integration of *bla*_CTX-M-65_ into the chromosome suggests that this drug resistance gene might be more resiliently maintained within the strains.

Appendix Additional information on chromosome-borne CTX-M-65 extended-spectrum β-lactamase–producing *Salmonella enterica* serovar Infantis, Taiwan.
