# Supplementary material for: Chromosome-Borne CTX-M-65 Extended-Spectrum β-Lactamase–Producing Salmonella enterica Serovar Infantis, Taiwan
Source: Emerg Infect Dis. 2023 Aug;29(8):1634–7. doi: 10.3201/eid2908.230472 (PMC10370839; doi:10.3201/eid2908.230472)
Supplement: Appendix — Additional information on chromosome-borne CTX-M-65 extended-spectrum β-lactamase–producing Salmonella enterica serovar Infantis, Taiwan. [file 23-0472-Techapp-s1.pdf]

Article DOI: https://doi.org/10.3201/eid2908.230472

EID cannot ensure accessibility for Supplemental Materials supplied by authors. Readers who have difficulty accessing supplementary content should contact the authors for assistance.

Chromosome-Borne CTX-M-65 Extended-Spectrum β-Lactamase–Producing *Salmonella enterica* Serovar Infantis, Taiwan

Appendix

Methods

Salmonella Isolates from Humans

Salmonella isolates recovered from human salmonellosis were obtained from collaborative hospitals across Taiwan from 2004 to 2022, under a disease surveillance project (PulseNet Taiwan). However, the collection of isolates was interrupted for 1 year in 2020. The Institutional Review Board of the Taiwan Centers for Disease Control (Taiwan CDC) approved the project, which waived the need for informed consent. Bacterial isolates from hospitals were re-confirmed for *Salmonella* using the MALDI Biotyper (Bruker Corp. USA). The *Salmonella* isolates were then genotyped by using the standardized PulseNet pulsed-field gel electrophoresis (PFGE) protocol (*1*), and their

serotypes were determined through PFGE pattern comparison with those in the *Salmonella* PFGE database established by Taiwan CDC (*2*).

***Salmonella* Isolates from Chickens**

To investigate the prevalence of $bla_{\text{CTX-M-65}}$-carrying *S.* Infantis strains in chickens, we collected 291 chicken meat samples from 12 supermarket stores in Taichung City from February to October 2022. All of the chicken meat samples were sourced from domestic farms. We followed standard procedures to isolate *Salmonella* from these samples. Briefly, we added 20 g chicken meat to 180 mL buffered peptone water (BPW) from Becton, Dickinson and Company (NJ, USA) and shook it at 25 rpm/min at room temperature for 5 min. We incubated and shook the mixture of BPW and meat at 100 rpm/min at 35 ± 2°C for 20–24 h. Next, we transferred 0.5 ml and 0.1 ml of the primary enrichment to 10 mL Tetrathionate Broth (TT broth) and Rappaport-Vassiliadis R10 broth (RV broth) from Becton, Dickinson and Company, respectively. We then incubated and shook the mixtures at 100 rpm/min at 42 ± 0.5°C for 20–24 h. We cultured a loop of secondary enrichment by streaking it on Xylose Lysine Tergitol-4 (XLT-4) plates from Becton, Dickinson and Company and incubated the plates at 35 ± 2°C for 18–24 h. Finally, we selected five typical colonies for each sample and identified them by using the MALDI Biotyper. We subjected one or two *Salmonella* isolates from each positive sample to PFGE analysis and determined their serotypes by comparing their PFGE patterns with those in the *Salmonella* PFGE database (*2*).

Salmonella Isolates from Pigs

We identified a single *S.* Infantis isolate from a group of 226 *Salmonella* isolates, which were recovered from pigs with illnesses at the Animal Disease Diagnostic Center of National Chiayi University between 2018 and 2022.

Salmonella PFGE Database

BioNumerics software version 6.6 (Applied Maths, Sint-Martens-Latem, Belgium) was used to establish and manage the *Salmonella* PFGE fingerprint database at the Taiwan CDC. The database included PFGE, serotypes, antimicrobial susceptibility testing (AST) results, and demographic data related to the source of *Salmonella* isolates. *Salmonella* isolates were collected from hospitals nationwide from 2004 to 2019 and 2021 to 2022 and the isolates were genotyped by using the PulseNet standardized protocol (*1*). For isolates collected from 2004 to 2006, serotypes were determined by using the conventional slide agglutination method with antisera purchased from S &A Reagents Laboratory (Bangkok, Thailand) (*3*). For the isolates collected from 2007 to 2019 and 2021 to 2022, serotypes were determined by using the PFGE pattern comparison approach (*2*). For those whose serotypes could not be determined by PFGE pattern comparison, flagella gene sequencing, and antisera agglutination were performed (*2*).

Antimicrobial Drug Susceptibility Testing

The AST was conducted by using the micro-broth dilution method and a custom-made 96-well Sensititer MIC panels (TREK Diagnostic Systems LTD., West Sussex, UK). The first MIC panel consisted of 12 antimicrobials: ampicillin, cefotaxime, ceftriaxone, nalidixic acid, ciprofloxacin, gentamicin, chloramphenicol, streptomycin,

sulfamethoxazole, cotrimoxazole (trimethoprim/sulfamethoxazole), trimethoprim, and tetracycline. However, the panel underwent several modifications, including the addition of ceftazidime and imipenem in 2005, the addition of cefoxitin, colistin, and ertapenem, and the removal of ceftriaxone and trimethoprim in 2010, the removal of imipenem in 2016, the addition of azithromycin in 2017, and the removal of cefoxitin and ertapenem in 2019. The latest AST panel used was Sensititer EUVSEC3, which consists of azithromycin, ampicillin, cefotaxime, ceftazidime, meropenem, nalidixic acid, ciprofloxacin, gentamicin, chloramphenicol, sulfamethoxazole, trimethoprim, tetracycline, colistin, and tigecycline. The test procedure adhered to the manufacturer's instructions, and the interpretation of MIC results followed the guidelines set by the Clinical and Laboratory Standards Institute or the ECAST standards (*4,5*).

Whole-Genome Sequencing

We used the Illumina MiSeq platform (Illumina Inc., California, USA) and the Oxford nanopore sequencing platform (Oxford Nanopore Technologies Limited, Oxford, UK) for whole-genome sequencing (WGS). A total of 51 *S.* Infantis isolates underwent Illumina sequencing, and seven of them were additionally sequenced by using the Oxford nanopore platform to obtain long sequence reads. The SPAdes assembler version 3.15.3 (*6*) was used for assembling sequence reads generated by the Illumina platform. For nanopore sequencing, Guppy v6.3.8 was used for basecalling, Flye v2.9.1 was used for assembly (*7*), and Medaka v1.7.2 (https://github.com/nanoporetech/medaka) was used for polishing. To enhance the accuracy of genome sequences, assembled nanopore sequences were polished with Illumina sequence reads by using Polypolish v0.5.0 (*8*) and POLCA (*9*).

Analysis of WGS Data

The cgMLST profiles were generated by using an in-house-developed BENGA cgMLST profiling tool and the *Salmonella* cgMLST scheme that comprises 3,265 core genes (https://github.com/openCDCTW/Benga). We used the AMRFinderPlus pipeline provided in the NCBI database (*10*), as well as ResFinder and PlamidFinder provided by the Center for Genomic Epidemiology (http://www.genomicepidemiology.org), to determine antimicrobial resistance genes, relevant resistance mutations, conventional sequence types (STs), and plasmid incompatibility types from the assembled contigs of each isolate.

Genetic Relatedness of Isolates Constructed by Using PFGE Patterns

The genetic relationships among isolates of *S.* Infantis were constructed by using clustering analysis of their PFGE patterns, using the tools provided by BioNumerics version 6.6 (Applied Maths). The similarity between two PFGE patterns was measured by using the Dice similarity coefficient with a setting of 1.5% optimization and 0.75% to 1.0% tolerance. The resulting PFGE tree (Appendix Figure) was generated by using the unweighted pair group method with arithmetic mean (UPGMA) algorithm, with a setting of 1.5% optimization and 1.0% tolerance.

Genetic Relatedness of Strains from Different Countries

To investigate the genetic relatedness of *bla*$_{\text{CTX-M-65}}$-carrying *S.* Infantis strains across different countries, we retrieved WGS data of strains from the National Center for Biotechnology Information database (https://www.ncbi.nlm.nih.gov/). Using the BENGA cgMLST profiling tool and the *Salmonella* cgMLST scheme (https://github.com/openCDCTW/Benga), we generated cgMLST profiles for these

strains. The cgMLST scheme, which comprises 3,265 core genes, was developed by using 715 genomes from various *Salmonella* serovars. The methods used in this scheme are outlined in a previous study (*11*). For our analysis, we selected 39 strains from 17 different countries and compared their genetic relatedness with 51 strains originating from Taiwan (detailed information on the strains can be found in Appendix Table 5). We used the UPGMA algorithm and the tools provided by BioNumerics version 7.6 (Applied Maths) to construct a cgMLST tree representing all 90 strains.

References

1. Ribot EM, Fair MA, Gautom R, Cameron DN, Hunter SB, Swaminathan B, et al. Standardization of pulsed-field gel electrophoresis protocols for the subtyping of *Escherichia coli* O157:H7, *Salmonella*, and *Shigella* for PulseNet. Foodborne Pathog Dis. 2006;3:59–67. PubMed https://doi.org/10.1089/fpd.2006.3.59

2. Chiou CS, Torpdahl M, Liao YS, Liao CH, Tsao CS, Liang SY, et al. Usefulness of pulsed-field gel electrophoresis profiles for the determination of *Salmonella* serovars. Int J Food Microbiol. 2015;214:1–3. PubMed https://doi.org/10.1016/j.ijfoodmicro.2015.07.016

3. Chiou CS, Huang JF, Tsai LH, Hsu KM, Liao CS, Chang HL. A simple and low-cost paper-bridged method for *Salmonella* phase reversal. Diagn Microbiol Infect Dis. 2006;54:315–7. PubMed https://doi.org/10.1016/j.diagmicrobio.2005.10.009

4. European Committee on Antimicrobial Susceptibility Testing. Breakpoint tables for interpretation of MICs and zone diameters version 11.0. http://wwweucastorg/clinical_breakpoints/ 2021.

5. CLSI. Performance standards for antimicrobial susceptibility testing. 30th ed. CLSI supplement M100. Wayne (PA): Clinical and Laboratory Standards Institute; 2020.

6. Bankevich A, Nurk S, Antipov D, Gurevich AA, Dvorkin M, Kulikov AS, et al. SPAdes: a new genome assembly algorithm and its applications to single-cell sequencing. J Comput Biol. 2012;19:455–77. PubMed https://doi.org/10.1089/cmb.2012.0021

7. Kolmogorov M, Yuan J, Lin Y, Pevzner PA. Assembly of long, error-prone reads using repeat graphs. Nat Biotechnol. 2019;37:540–6. PubMed https://doi.org/10.1038/s41587-019-0072-8

8. Wick RR, Holt KE. Polypolish: Short-read polishing of long-read bacterial genome assemblies. PLOS Comput Biol. 2022;18:e1009802. PubMed https://doi.org/10.1371/journal.pcbi.1009802

9. Zimin AV, Salzberg SL. The genome polishing tool POLCA makes fast and accurate corrections in genome assemblies. PLOS Comput Biol. 2020;16:e1007981. PubMed https://doi.org/10.1371/journal.pcbi.1007981

10. Feldgarden M, Brover V, Fedorov B, Haft DH, Prasad AB, Klimke W. Curation of the AMRFinderPlus databases: applications, functionality and impact. Microb Genom. 2022;8:mgen000832. PubMed https://doi.org/10.1099/mgen.0.000832

11. Liu YY, Chen CC, Chiou CS. Construction of a pan-genome allele database of *Salmonella enterica* serovar Enteritidis for molecular subtyping and disease cluster identification. Front Microbiol. 2016;7:2010. PubMed https://doi.org/10.3389/fmicb.2016.02010

Appendix Table 1. Antimicrobial resistance in *bla*$_{CTX-M-65}$-carrying *S.* Infantis isolates and non- *bla*$_{CTX-M-65}$-carrying isolates

	blaCTX-M-65-carrying clone*		Others†	
Antimicrobial	No. isolates	Resistance, %	No. isolates	Resistance, %
Azithromycin	47	2.1	60	0
Ampicillin	47	100	148	4.1
Cefotaxime	47	100	148	2.7
Ceftazidime	47	100	137	2.9
Meropenem	47	0	26	3.8
Nalidixic acid	47	100	148	2.0
Ciprofloxacin	47	0	148	0
CiprofloxacinRS‡	47	100	148	2.7
Gentamicin	47	17.0	148	0.7
Chloramphenicol	47	93.6	148	2.7
Sulfamethoxazole	47	100	148	15.5
Trimethoprim	47	89.4	84	19.0
Tetracycline	47	100	148	2.0
Colistin	47	0	90	0
Tigecycline	47	0	26	0

**S.* Infantis isolates recovered in humans and chicken meat in 2021 and 2022.

†*S.* Infantis isolates recovered from humans from 2004 to 2019.

‡RS, reduced susceptibility.

Appendix Table 2. PFGE patterns for *bla*$_{CTX-M-65}$-carrying *S.* Infantis isolates

	S. Infantis isolates from:			
PFGE pattern	Human	Chicken	Pig	Total
SMX.1767	15	167		182
SMX.1821	5	14	1	20
SMX.1819	1	11		12
SMX.1818	1	10		11
SMX.1816	2	6		8
SMX.1827		7		7
SMX.1890		5		5
SMX.1823		5		5

PFGE pattern	S. Infantis isolates from: Human	Chicken	Pig	Total
SMX.1824	1	3		4
SMX.1862		3		3
SMX.1919		2		2
SMX.1920		2		2
SMX.1866		2		2
SMX.1921		2		2
SMX.1918		2		2
SMX.1820		2		2
SMX.1811		2		2
SMX.1825		2		2
SMX.1828		2		2
SMX.1822		1		1
SMX.1791		1		1
SMX.1834		1		1
SMX.1863		1		1
SMX.1870	1			1
SMX.1817		1		1
SMX.1833	1			1
SMX.1867		1		1
SMX.1861		1		1
SMX.1868		1		1
SMX.1869		1		1
SMX.1832	1			1
Total	28	258	1	287

A**ppendix Table 3.** Sources, genetic determinants of resistance, and plasmid incompatibility types of *S.* Infantis isolates that have undergone whole-genome sequencing

Isolate ID	SRA accession	Source Type	Isolate Year	ST	Antimicrobial resistance gene	Resistance relevant mutation	Plasmid type
NC04.268	ERR2759825	Human	2004	32			
NH04.059	ERR2759750	Human	2004	32	*dfrA1, mph(A), sul1*		Col(pHAD28)
NL04.149	ERR4399149	Human	2004	32			
SG04.129	ERR2759834	Human	2004	32			
NC05.302	ERR2719627	Human	2005	32	*dfrA1, mph(A), sul1*		
NC05.438	SRR13853524	Human	2005	32			
CE06.086	ERR2719633	Human	2006	32	*dfrA1, mph(A), sul1*		
NG06.087	SRR13853512	Human	2006	32			
CC07.003	ERR2719639	Human	2007	32			
NA07.065	ERR2719657	Human	2007	32			
NC07.024	ERR4400838	Human	2007	32			
CE08.063	ERR4338231	Human	2008	32			
NJ09.293	SRR13853503	Human	2009	32			
EB13.001	SRR8196858	Human	2013	32	bla_{CMY-2}		Col(pHAD28), Col440II, ColRNAI, IncFIC(FII), IncFII(SARC14), IncFII(p14), IncI1-I(α), IncL
SD14.037	SRR23864774	Human	2014	32			
R15.2426	SRR23864785	Human	2015	32			
R16.4088	SRR23864759	Human	2016	32			
R16.5055	SRR13853522	Human	2016	32			
R17.0992	SRR13853521	Human	2017	32			
R17.1199	SRR23864765	Human	2017	32			
R17.1683	SRR23864775	Human	2017	32			
R17.2860	SRR23864776	Human	2017	32			Col8282, IncI1-I(α)
R17.3860	SRR23864777	Human	2017	32			

Isolate ID	SRA accession	Source Type	Isolate Year	ST	Antimicrobial resistance gene	Resistance relevant mutation	Plasmid type
R17.5425	SRR8189531	Human	2017	32	*aadA1, bla*$_{CTX\text{-}M\text{-}14}$*, dfrA14, sul1, tet(A)*	*gyrA*_D87Y	IncFIB, IncX4
R18.0852	SRR13853507	Human	2018	32			
R18.0926	SRR23864778	Human	2018	32			
R18.0993	SRR23864783	Human	2018	32			
R18.1364	SRR13853506	Human	2018	32			
R18.1512	SRR13853505	Human	2018	32			
R18.1553	SRR13853504	Human	2018	32			
R21.0914	SRR23864758	Human	2021	32	*aac(3)-IVa, aadA1, aph(3')-Ia, aph(4)-Ia, bla*$_{CTX\text{-}M\text{-}65}$*, dfrA14, floR, sul1, tet(A)*	*gyrA*_D87Y	IncFIB
R21.0919	SRR23864757	Human	2021	32			
R21.1147	SRR18297517	Human	2021	32	*aac(3)-IVa, aadA1, aph(3')-Ia, aph(4)-Ia, bla*$_{CTX\text{-}M\text{-}65}$*, dfrA14, floR, sul1, tet(A)*	*gyrA*_D87Y	IncFIB
R21.1547	SRR23864760	Human	2021	32			
R21.1575	SRR23864779	Human	2021	32	*aac(3)-IVa, aadA1, aph(3')-Ia, aph(4)-Ia, bla*$_{CTX\text{-}M\text{-}65}$*, dfrA14, floR, sul1, tet(A)*	*gyrA*_D87Y	IncFIB
R22.0038	SRR23864780	Chicken	2022	32	*aac(3)-IVa, aadA1, aph(3')-Ia, aph(4)-Ia, bla*$_{CTX\text{-}M\text{-}65}$*, dfrA14, floR, sul1, tet(A)*	*gyrA*_D87Y	IncFIB
R22.0040	SRR23864781	Chicken	2022	32	*aac(3)-IVa, aadA1, aph(3')-Ia, aph(4)-Ia, bla*$_{CTX\text{-}M\text{-}65}$*, dfrA14, floR, sul1, tet(A)*	*gyrA*_D87Y	IncFIB
R22.0042	SRR23864782	Chicken	2022	32	*aac(3)-IVa, aadA1, aph(3')-Ia, aph(4)-Ia, bla*$_{CTX\text{-}M\text{-}65}$*, dfrA14, floR, sul1, tet(A)*	*gyrA*_D87Y	IncFIB
R22.0044	SRR23864784	Chicken	2022	32	*aac(3)-IVa, aadA1, aph(3')-Ia, aph(4)-Ia, bla*$_{CTX\text{-}M\text{-}65}$*, bla*$_{TEM}$*, dfrA14, floR, sul1, tet(A)*	*gyrA*_D87Y	IncFIB, IncQ1
R22.0223	SRR23864761	Chicken	2022	32	*aadA1, bla*$_{CTX\text{-}M\text{-}65}$*, sul1, tet(A)*	*gyrA*_D87Y	IncFIB

Isolate ID	SRA accession	Source Type	Isolate Year	ST	Antimicrobial resistance gene	Resistance relevant mutation	Plasmid type
R22.1093	SRR23864762	Chicken	2022	32	*aac(3)-IVa, aadA1, aph(3')-Ia, aph(4)-Ia, bla*$_{CTX-M-65}$, *dfrA14, floR, sul1, tet(A)*	*gyrA*_D87Y	IncFIB
R22.1155	SRR23864763	Chicken	2022	32	*aac(3)-IVa, aadA1, aph(3')-Ia, aph(4)-Ia, bla*$_{CTX-M-65}$, *dfrA14, sul1, tet(A)*	*gyrA*_D87Y	IncFIB
R22.1428	SRR23864764	Chicken	2022	32	*aac(3)-IVa, aadA1, aph(3')-Ia, aph(4)-Ia, bla*$_{CTX-M-65}$, *dfrA14, floR, sul1, tet(A)*	*gyrA*_D87Y	IncFIB
R22.1516	SRR23864766	Chicken	2022	32	*aac(3)-IVa, aadA1, aph(3')-Ia, aph(4)-Ia, bla*$_{CTX-M-65}$, *dfrA14, floR, sul1, tet(A)*	*gyrA*_D87Y	IncFIB
R22.1900	SRR23864767	Chicken	2022	32	*aac(3)-IVa, aadA1, aph(3')-Ia, aph(4)-Ia, bla*$_{CTX-M-65}$, *dfrA14, floR, sul1, tet(A)*	*gyrA*_D87Y	IncFIB
R22.2169	SRR23864768	Chicken	2022	32	*aac(3)-IVa, aadA1, aph(3')-Ia, aph(4)-Ia, bla*$_{CTX-M-65,}$ *dfrA14, floR, sul1, tet(A)*	*gyrA*_D87Y	IncFIB
R22.2281	SRR23864769	Chicken	2022	32	*aac(3)-IVa, aadA1, aph(3')-Ia, aph(4)-Ia, bla*$_{CTX-M-65}$, *floR, sul1, tet(A)*	*gyrA*_D87Y	IncFIB
R22.2574	SRR23864770	Human	2022	32			
R22.2646	SRR23864771	Chicken	2022	32	*aac(3)-IVa, aadA1, aph(3')-Ia, aph(4)-Ia, bla*$_{CTX-M-65}$, *dfrA14, floR, sul1, tet(A)*	*gyrA*_D87Y	IncFIB
R22.3078	SRR23864772	Chicken	2022	32	*aac(3)-IVa, aadA1, aph(3')-Ia, aph(4)-Ia, bla*$_{CTX-M-65}$, *dfrA14, floR, sul1, tet(A)*	*gyrA*_D87Y	IncFIB
R22.3740	SRR23864773	Pig	2022	32	*aac(3)-IVa, aadA1, aph(3')-Ia, aph(4)-Ia, bla*$_{CTX-M-65}$, *dfrA14, floR, sul1, tet(A)*	*gyrA*_D87Y	IncFIB

Appendix Table 4. Characteristics of seven *S.* Infantis isolates with complete genomes sequences

Isolate ID	Source Type	Isolate Year	Chromosome, bp (accession no.)	IncFIB plasmid, bp (accession no.)	ARGs in chromosome*	ARG in IncFIB plasmid
R21.0914	Human	2021	4,850,920 (CP121066.1)	195,329 (CP121067.1)	*aadA1, bla*$_{CTX-M-65}$, *sul1, tet(A)*	*aac(3)-IVa, aph(3')-Ia, aph(4)-Ia, dfrA14, floR*
R21.1147	Human	2021	4,851,135 (CP093400.1)	195,315 (CP093401.1)	*aadA1, bla*$_{CTX-M-65}$, *sul1, tet(A)*	*aac(3)-IVa, aph(3')-Ia, aph(4)-Ia, dfrA14, floR*
R21.1575	Human	2021	4,857,909 (CP121068.1)	195,307 (CP121069.19)	*aadA1, bla*$_{CTX-M-65}$, *sul1, tet(A)*	*aac(3)-IVa, aph(3')-Ia, aph(4)-Ia, dfrA14, floR*
R22.0044	Chicken	2022	4,851,681 (CP121070.1)	195,305 (CP121071.1)	*aadA1, bla*$_{CTX-M-65}$, *sul1, tet(A)*	*aac(3)-IVa, aph(3')-Ia, aph(4)-Ia, bla*$_{TEM}$, *dfrA14, floR*
R22.2169	Chicken	2022	4,851,438 (CP121073.1)	195,026 (CP121074.1)	*aadA1, bla*$_{CTX-M-65}$, *sul1, tet(A)*	*aac(3)-IVa, aph(3')-Ia, aph(4)-Ia, dfrA14, floR*
R22.2574	Human	2022	4,679,202 (CP121075.1)			
R22.3740	Pig	2022	4,851,036 (CP121076.1)	195,315 (CP121077.1)	*aadA1, bla*$_{CTX-M-65}$, *sul1, tet(A)*	*aac(3)-IVa, aph(3')-Ia, aph(4)-Ia, dfrA14, floR*

*ARG, antimicrobial resistance gene.

Appendix Table 5. *S.* Infantis strains for constructing the cgMLST genetic tree in Figure 1

Strain ID	Seq Source ID	Isolate Year	Source Country	Source Type	Biosample	Note
N16S097	GCA_009584525.2	2016	USA	Poultry	SAMN08113976	
287724	GCA_009142785.1	2016	Bolivia	Human	SAMN06248021	
532490	GCA_007507545.1	2018	Brazil	Poultry	SAMN10848643	
531219	GCA_007271025.1	2018	Chile	Poultry	SAMN09638051	
506074	GCA_006984665.1	2018	Chile	Poultry	SAMN09634250	
591831	GCA_007293015.1	2018	Dominican Republic	Human	SAMN10067791	
hPHE_95	GCA_018841615.1	2013	Ecuador	Human	SAMN19652534	
561097	GCA_007652195.1	2018	India	Human	SAMN10835753	
hPHE_169	GCA_018842575.1	2013	Peru	Human	SAMN19652538	
93556	GCA_008071765.1	2015	Peru	Human	SAMN03476638	
385895	GCA_009568345.1	2017	Peru	Human	SAMN07946468	
43931	GCA_008739615.1	2014	Peru	Human	SAMN04363560	
617907	GCA_007328735.1	2018	Spain	Human	SAMN10229167	
Se_Q_005	GCA_020867825.1	2018	Ecuador	human	SAMEA7302379	
19–02053	GCA_020159045.1	2019	Germany	Human	SAMEA7540898	
18–05623	GCA_020159425.1	2018	Germany	Human	SAMEA7540870	
18–01981	GCA_020142245.1	2018	Germany	Human	SAMEA7540841	
19–03947	GCA_020158705.1	2019	Germany	Human	SAMEA7540917	
18–00383	GCA_020159945.1	2018	Germany	Human	SAMEA7540830	
2013AM-1918	GCA_006907145.1	2013	USA	Human	SAMN05570537	
N55391	GCA_001931595.1	2014	USA	Poultry	SAMN03988471	
FSIS1502973	GCA_008649995.1	2015	USA	Cow	SAMN04285054	
FSIS1502169	GCA_001931555.1	2015	USA	Poultry	SAMN04285042	
14026835	GCA_010797855.1	2014	Italy	Human	SAMEA3539004	
13–3055	GCA_024984095.1	2013	Canada	Human	SAMN19836089	
15–4113	GCA_024984075.1	2015	Canada	Human	SAMN19836107	
14–7715	GCA_024984195.1	2014	Canada	Human	SAMN19836100	
16–2005	GCA_024983495.1	2016	Canada	Human	SAMN19836111	
UZH-SAL-144–13	GCA_002200365.1	2013	Switzerland	Poultry	SAMN06552091	
UZH-SAL-125–15	GCA_002200405.1	2015	Switzerland	Human	SAMN06552094	

Strain ID	Seq Source ID	Isolate Year	Source Country	Source Type	Biosample	Note
UZH-SAL-21–13	GCA_002200555.1	2013	Switzerland	Human	SAMN06552077	
TTU_312	GCA_003917175.1	2017	Colombia	Poultry	SAMN10129553	
Se_Q_151	GCA_019814545.1	2018	Ecuador	Poultry	SAMEA7302514	
MOD1_Per24	GCA_006211825.1	2011	Peru	Human	SAMN07244637	
MOD1_Per141	GCA_004187315.1	2012	Peru	Human	SAMN08225178	
AUSMDU00009672	GCA_017799155.1	2017	Australia	Human	SAMN18618485	
NG06.087	SRR13853512	2006	Taiwan	Human	SAMN18133167	This study
R16.5055	SRR13853522	2016	Taiwan	Human	SAMN18133175	This study
NC07.024	ERR4400838	2007	Taiwan	Human	SAMEA7114331	This study
CE08.063	ERR4338231	2008	Taiwan	Human	SAMEA5670929	This study
SD14.037	SRR23864774	2014	Taiwan	Human	SAMN33760355	This study
R15.2426	SRR23864785	2015	Taiwan	Human	SAMN33760327	This study
R18.1553	SRR13853504	2018	Taiwan	Human	SAMN18133192	This study
R21.0919	SRR23864757	2021	Taiwan	Human	SAMN33760336	This study
R17.1683	SRR23864775	2017	Taiwan	Human	SAMN33760330	This study
NC05.438	SRR13853524	2005	Taiwan	Human	SAMN18133165	This study
R16.4088	SRR23864759	2016	Taiwan	Human	SAMN33760328	This study
R18.1512	SRR13853505	2018	Taiwan	Human	SAMN18133191	This study
R17.0992	SRR13853521	2017	Taiwan	Human	SAMN18133176	This study
R17.3860	SRR23864777	2017	Taiwan	Human	SAMN33760332	This study
R17.2860	SRR23864776	2017	Taiwan	Human	SAMN33760331	This study
R18.0993	SRR23864783	2018	Taiwan	Human	SAMN33760334	This study
NA07.065	ERR2719657	2007	Taiwan	Human	SAMEA104279689	This study
R18.0852	SRR13853507	2018	Taiwan	Human	SAMN18133189	This study
R18.0926	SRR23864778	2018	Taiwan	Human	SAMN33760333	This study
NL04.149	ERR4399149	2004	Taiwan	Human	SAMEA7112639	This study
NH04.059	ERR2759750	2004	Taiwan	Human	SAMEA104103372	This study
NC05.302	ERR2719627	2005	Taiwan	Human	SAMEA104279673	This study
CE06.086	ERR2719633	2006	Taiwan	Human	SAMEA104279680	This study
EB13.001	SRR8196858	2013	Taiwan	Human	SAMN10434137	This study
R17.5425	SRR8189531	2017	Taiwan	Human	SAMN10425365	This study
R17.1199	SRR23864765	2017	Taiwan	Human	SAMN33760329	This study
R21.1547	SRR23864760	2021	Taiwan	Human	SAMN33760337	This study

Strain ID	Seq Source ID	Isolate Year	Source Country	Source Type	Biosample	Note
CC07.003	ERR2719639	2007	Taiwan	Human	SAMEA104279697	This study
R22.1093	SRR23864762	2022	Taiwan	Poultry	SAMN33760344	This study
R22.3078	SRR23864772	2022	Taiwan	Poultry	SAMN33760353	This study
R22.3740	SRR23864773	2022	Taiwan	Pig	SAMN33760354	This study
R22.1516	SRR23864766	2022	Taiwan	Poultry	SAMN33760347	This study
R22.1900	SRR23864767	2022	Taiwan	Poultry	SAMN33760348	This study
R22.2281	SRR23864769	2022	Taiwan	Poultry	SAMN33760350	This study
R21.0914	SRR23864758	2021	Taiwan	Human	SAMN33760335	This study
R21.1147	SRR18297517	2021	Taiwan	Human	SAMN26567210	This study
R22.2169	SRR23864768	2022	Taiwan	Poultry	SAMN33760349	This study
R22.0038	SRR23864780	2022	Taiwan	Poultry	SAMN33760339	This study
R22.0040	SRR23864781	2022	Taiwan	Poultry	SAMN33760340	This study
R22.0223	SRR23864761	2022	Taiwan	Poultry	SAMN33760343	This study
R22.2646	SRR23864771	2022	Taiwan	Poultry	SAMN33760352	This study
R21.1575	SRR23864779	2021	Taiwan	Human	SAMN33760338	This study
R22.0042	SRR23864782	2022	Taiwan	Poultry	SAMN33760341	This study
R22.1428	SRR23864764	2022	Taiwan	Poultry	SAMN33760346	This study
R22.1155	SRR23864763	2022	Taiwan	Poultry	SAMN33760345	This study
R22.0044	SRR23864784	2022	Taiwan	Poultry	SAMN33760342	This study
SG04.129	ERR2759834	2004	Taiwan	Human	SAMEA104103459	This study
NJ09.293	SRR13853503	2009	Taiwan	Human	SAMN18133168	This study
NC04.268	ERR2759825	2004	Taiwan	Human	SAMEA104103450	This study
R18.1364	SRR13853506	2018	Taiwan	Human	SAMN18133190	This study
R22.2574	SRR23864770	2022	Taiwan	Human	SAMN33760351	This study
24990	GCA_008098565.1	2014	United Kingdom	Human	SAMN03477912	
VNSEC002	GCA_013377335.1	2017	Vietnam	Poultry	SAMN11355817	
VNSEC001	GCA_013377355.1	2017	Vietnam	Poultry	SAMN11355816	

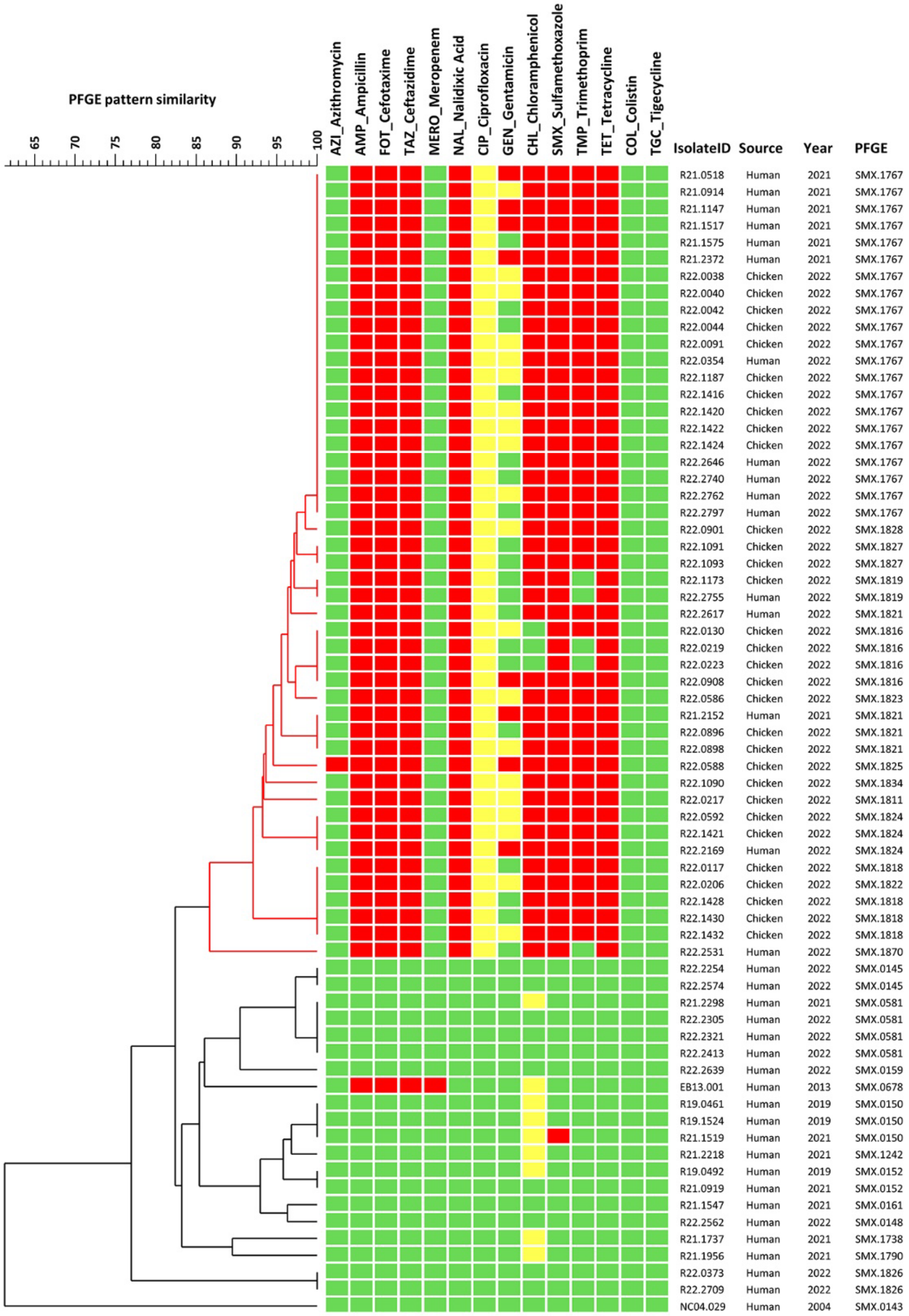

Appendix Figure. Genetic relatedness among *S.* Infantis isolates based on PFGE patterns. Clustering analysis was performed on isolates with available AST data determined using the latest testing panel, Sensititer® EUVSEC3 kit. The antimicrobial susceptibility testing results are

depicted using colored squares, with red indicating resistance, yellow indicating intermediate susceptibility, and green indicating susceptibility. The highlighted red cluster comprises multidrug-resistant *bla*$_{CTX\text{-}M\text{-}65}$-carrying isolates recovered from both human and chicken meat samples in 2021 and 2022.
